# Orbitofrontal Cortex Functional Connectivity-Based Classification for Chronic Insomnia Disorder Patients With Depression Symptoms

**DOI:** 10.3389/fpsyt.2022.907978

**Published:** 2022-07-06

**Authors:** Liang Gong, Ronghua Xu, Dan Yang, Jian Wang, Xin Ding, Bei Zhang, Xingping Zhang, Zhengjun Hu, Chunhua Xi

**Affiliations:** ^1^Department of Neurology, Chengdu Second People’s Hospital, Chengdu, China; ^2^Department of General Practice, Chengdu Second People’s Hospital, Chengdu, China; ^3^The Third People’s Hospital of Chengdu, Chengdu, China; ^4^Department of Neurology, The Third Affiliated Hospital of Anhui Medical University, Hefei, China

**Keywords:** insomnia, depression, orbitofrontal cortex, functional connectivity, machine learning

## Abstract

Depression is a common comorbid symptom in patients with chronic insomnia disorder (CID). Previous neuroimaging studies found that the orbital frontal cortex (OFC) might be the core brain region linking insomnia and depression. Here, we used a machine learning approach to differentiate CID patients with depressive symptoms from CID patients without depressive symptoms based on OFC functional connectivity. Seventy patients with CID were recruited and subdivided into CID with high depressive symptom (CID-HD) and low depressive symptom (CID-LD) groups. The OFC functional connectivity (FC) network was constructed using the altered structure of the OFC region as a seed. A linear kernel SVM-based machine learning approach was carried out to classify the CID-HD and CID-LD groups based on OFC FC features. The predict model was further verified in a new cohort of CID group (*n* = 68). The classification model based on the OFC FC pattern showed a total accuracy of 76.92% (*p* = 0.0009). The area under the receiver operating characteristic curve of the classification model was 0.84. The OFC functional connectivity with reward network, salience network and default mode network contributed the highest weights to the prediction model. These results were further validated in an independent CID group with high and low depressive symptom (accuracy = 67.9%). These findings provide a potential biomarker for early diagnosis and intervention in CID patients comorbid with depression based on an OFC FC-based machine learning approach.

## Introduction

Chronic insomnia disorder (CID) is defined by patient dissatisfaction with sleep quality or duration (with both nighttime insomnia symptoms and daytime insomnia symptoms) and is present for more than three nights per week and lasts for 3 months (1). The prevalence rate of CID is approximately 10% worldwide, and insomnia is associated with significant direct and indirect costs (2). In addition, insomnia is commonly associated with mental disorders, such as anxiety and depression. The chronic nature of insomnia and its associated mental symptoms, especially depression, could profoundly burden the quality of life and even increase the suicide rate (3–5). Previous studies show that insomnia patients have a 3–4 times higher risk of major depressive disorder than the normal population (6). Despite the high prevalence of CID and its substantial influence on the quality of life and disease burden, the exact neural mechanism of CID combined with depression is not clear. On the other hand, insomnia is a heterogeneous disorder with different clinical phenotypes, such as CID with higher depressive and lower depressive symptoms (7). Different clinical phenotypes are attributed to different neuropathological mechanisms (8) and contribute to different treatment responses (9). Thus, there is a substantial need to elucidate the underlying pathophysiology and useful biomarkers of CID comorbid depressive symptoms.

Recently, advanced neuroimaging technology and machine learning have been combined to explore potential biomarkers of neuropsychiatric disorders, such as chronic pain (10, 11), Alzheimer’s disease (12–14), schizophrenia (15), major depressive disorder (MDD), and insomnia disorder (16). Li et al. used a support vector machine (SVM) combined with functional MRI data and found that functional connectivity (FC) strength might identify primary insomnia from healthy controls, and FC in the anterior insular cortex and left middle frontal gyrus showed high classification weights in the classifier (16). However, no study has explored brain biomarkers in a subgroup of CID patients with depressive symptoms.

The orbitofrontal cortex (OFC) is the critical brain area in emotion and reward value processing (17). Abnormal OFC structure and function might underlie the pathophysiology of mental disorders, such as depression and anxiety (18, 19). Patients with insomnia also reported a small gray matter volume in the OFC, and an altered OFC volume was associated with the severity of insomnia (20, 21). For the interaction between insomnia and depression, our previous study identified that the right OFC gray matter was affected by the interaction of insomnia and depression (7). Cheng et al. also reported that lateral OFC functional connectivity increased in both higher sleep and depressive problem scores in 1017 healthy populations in the Human Connectome Project (HCP), and the OFC functional connectivity could mediate the relationship between sleep quality and depressive problem scores (22). More recently, the results from 1053 MDD patients in the ENIGMA dataset also found a smaller cortical surface area in the right OFC in MDD patients with more severe insomnia symptoms (23). However, whether the OFC feature could be used as a biomarker to detect depression symptom in CID patients have not been explored.

In the present study, we first investigated the potential OFC gray matter volume alteration in CID patients with high depressive symptoms (CID-HD) compared to CID patients with low depressive symptoms (CID-LD). Second, the altered OFC was employed to construct the OFC FC network in each group. Third, a linear kernel SVM-based machine learning approach was carried out to classify the CID-HD and CID-LD groups based on OFC FC features. Lastly, an independent cohort of CID was used to validate the machine learning results. According to the findings of our previous study and neuroimaging data of healthy and MDD participants, we hypothesized that the combined OFC structure and function features could discriminate CID-HD from CID-LD in CID patients.

## Materials and Methods

### Participants

Two cohorts of CID patients were used in the present study. The first cohort was seventy patients with CID, which were recruited from outpatients of the Department of Neurology at the Chengdu Second People’s Hospital (CSPH). The second cohort was sixty-eight patients with CID enrolled in the Third Affiliated Hospital of Anhui Medical University (AHMU), which was used to validate the results obtained from cohort one. The Research Ethics Committee of CSPH and AHMU approved this study [the Ethics number are 2020021 (CSPH) and 2019-010-1 (AHMU)], and all participants gave written informed consent. The inclusion criteria for CID were as follows: (1) diagnostic criteria for CID according to the 3rd version of the International Classification of Sleep Disorders (1); (2) at least 3 months of difficulty falling asleep, maintaining sleep, or early wakening; (3) absence of hypnotic or antidepressant medication in the 2 weeks before neuropsychological testing and MRI scan; and (4) age of 18–55 years, with age of insomnia onset under 50 years. The exclusion criteria for the CID group were as follows: (1) history of another neuropsychiatric disorder, such as major depressive disorder or general anxiety; (2) other sleep disorders, such as sleep-related breathing disorders (sleep apnea syndrome), central disorders of hypersomnolence, circadian rhythm sleep-wake disorders, sleep-related movement disorders, parasomnia, and hypersomnia [evaluated by two experienced neurologists (JW and CHX)]; (3) abuse of substances such as caffeine and alcohol; (4) contraindications to MRI; and (5) brain lesions identified using conventional MRI sequences.

### Behaviors Assessment and Subgroup Divide

All participants underwent a clinical assessment, and a neuropsychiatric examination performed by two experienced neurologists and consensus diagnoses were reached. The Pittsburgh Sleep Quality Index (PSQI) was employed to evaluate insomnia severity in the CID group (24, 25). The self-rating depression scale (SDS) was used for depression evaluation, and the self-rating anxiety scale (SAS) was used for anxiety evaluation (26, 27). According to our previous study (7), we used the SDS scale for CID subgroup deviation. According to the SDS score, in the first cohort, 26 patients with CID were enrolled in the CID with high depression symptoms (CID-HD, SDS score above 55), 26 patients with CID were enrolled in the CID with low depressive symptoms group (CID-LD, SDS score below 50), and 14 patients whose SDS score was between 50–55 were not included in the statistical analysis (28). In the second cohort, fifteen CID patients whose SDS scores was between 50 and 55 were not included in the next analysis. Finally, 24 CID-LD and 29 CID-HD patients in the second cohort were used for validating of the result of the cohort one.

### Image Acquisition

To avoid the influence of cortisol rhythm on brain functional connectivity, all the MRI was scanned between 4 p.m. and 6 p.m. (29) in all participants. Imaging was performed using a 3.0-Tesla MRI scanner (GE Health care Discovery MR750, Milwaukee, WI, United States) equipped with an 8-channel head coil. Structural images were acquired using high-resolution spoiled gradient-recalled echo with the following parameters: repetition time = 2900; echo time = 2.48 ms; flip angle = 7°; acquisition matrix = 256 × 256; field of view = 256 mm × 256 mm; thickness = 1.0 mm (with no gap); number of slices = 188; voxel size = 1 mm × 1 mm × 1 mm. The rs-fMRI datasets were obtained using an 8-min gradient-recalled echo-planar imaging pulse sequence with the following parameters: TR/TE = 2,000/35 ms; FA = 90°; acquisition matrix = 64 × 64; thickness = 3.5 mm; number of slices = 36, time points = 240 scans. During scanning, all participants were instructed to relax and keep their eyes closed, and stabilizers were used to immobilize the head. Wakefulness was assessed following scanning, and all participants claimed to be awake during the study.

### Image Preprocessing

The structural MRI data were preprocessed using the optimized voxel-based morphometry (VBM 8) toolbox in SPM12 implemented in MATLAB 8.0 (The MathWorks, Inc., Natick, MA, United States). First, the T1-weighted images were reoriented following the AC-PC line; Second, the reoriented images were segmented to gray matter (GM), white matter (WM) and cerebrospinal fluid (CSF); Third, the segmented GM was normalized to the standard Montreal Neurological Institute (MNI) space using the DARTEL algorithm (30), and the normalized images were smoothed with a Gaussian kernel (FWHM = 6 mm). The rs-fMRI data were preprocessed using SPM12 and DPABI 4.3 (Data Processing & Analysis of Brain Imaging) implemented in MATLAB 8.0 (31). The first ten initial volumes were discarded to account for the magnetization equilibrium and adaptation to the experimental environment. The remaining 230 images were then slice-time corrected, reoriented, realigned, and coregistered to the T1-weighted structural images, which were segmented using DARTEL (30). Images from all participants were normalized into standard stereotactic MNI space and smoothed using a Gaussian kernel (FWHM = 6 mm). Voxel time series were further detrended and temporally filtered (0.01–0.08 Hz). We normalized the variance of each time series to control for fluctuations in signal intensity. Noise associated with WM/CSF signals and 24 head motion-related covariates were regressed out. The global signal was not regressed out given the controversy regarding its application to rs-fMRI data (32, 33). Participants with head motion exceeding 2 mm or 2° were excluded for the imaging analysis.

### Demographic and Behavioral Analyses

Two-sample *t*-tests and chi-square tests (for sex comparison) were conducted to compare the demographic data between two groups (SPSS 20, Inc., Chicago, IL, United States). Pearson correlation was employed to examine the relationships between PSQI, SAS, SDS scores, and duration of disease in the CID group. The statistical significance was set at *p* < 0.05.

### Image Statistical Analysis

**FIGURE 1:**
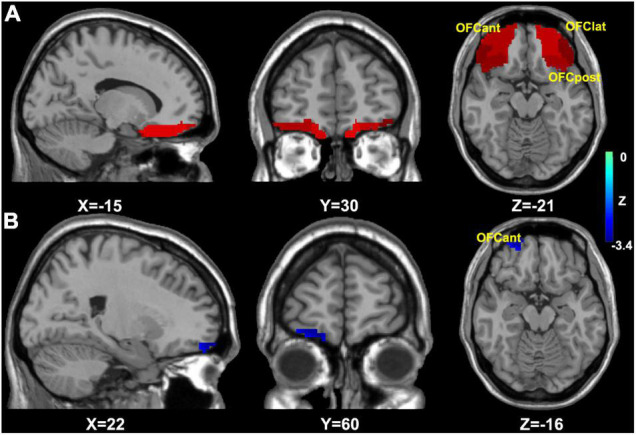
The mask and results of OFC based VBM analysis. **(A)** The OFC analysis mask for VBM analysis; **(B)** the group difference of OFC based VBM analysis, then the result (right OFCant) was selected for OFC FC analysis. OFC, orbital frontal cortex; OFCmed, medial OFC; OFCant, anterior OFC; OFClat, lateral OFC; OFCpost, posterior OFC; FC, functional connectivity.

First, as the OFC has been shown to link sleep and depression in previous studies (7, 34), we selected all OFC subregions as regions of interest from AAL 3 (35). Then, a voxelwise-based two-sample *t*-test was conducted to explore the structural difference between the CID-HD and CID-LD groups in the orbital frontal cortex, with age, sex, and total intracranial volume (TIV) as nuisance covariates. The voxel-level significance threshold was set at *p* < 0.005, which was corrected for multiple comparisons by using Gaussian random field (GRF) correction (*p* < 0.05) in the OFC mask (Figure 1A).

Second, as we found that the group difference in OFC volume was located in the right anterior OFC (see the result, Figure 1B), the right anterior OFC was selected as a seed based on the construction of the OFC FC network using DPABI. The averaged time course in the right anterior OFC was extracted and correlated with the time courses for all brain voxels using Pearson’s correlation analysis, and Fisher’s Z-transformation was applied to improve the correlation coefficient values to approach a normal distribution (36).

Third, a voxel-wise base two-sample *t*-test was conducted to acquire the group differences in the OFC FC network between two groups, with age and sex as nuisance covariates. The voxel level significance threshold was set at *p* < 0.05, which was corrected for multiple comparisons by using GRF correction (*p* < 0.05).

### Support Vector Machine Based Machine Learning Analysis

A linear kernel SVM analysis was carried out in PRoNTo_2.1.3 software (Pattern Recognition for Neuroimaging Toolbox) (37). We applied different significance thresholds (*p* < 0.01 and *p* < 0.05) in feature selection, corresponding to small and large numbers of features (38). Then, the individual OFC FC maps within the mask of group differences served as inputs for the machine learning algorithm. In each of the analyses, the linear kernel SVM soft margin parameter C was set to the default value (one) (39), and leaving one subject out was performed for cross-validation (LOOCV). The statistical significance of the accuracy of the classifications was tested using permutation testing with 1000 permutations with random assignment of group class to input image, using *p* < 0.05. The area under the receiver operating characteristic curve (AUC) was reported as the measure of accuracy. For the SVM features, the corresponding weight was computed in each voxel and summarized the weights of each region of interest (ROI) defined by the AAL 3 atlas (40).

To evaluate the generalizability of the OFC FC-based classifier, we applied the trained classifier to an independent cohort of CID group with high and low depressive symptoms with the same scanned parameters and preprocessing pipeline. In the verified analysis, dataset 2 set as the predicted sample.

## Results

### Demographic Information and Clinical Features

Seventy patients (42 female) with CID enrolled and completed all the clinical evaluation and MRI scans. According to the SDS score, the CID was subdivided into CID-HD (26 subjects) and CID-LD (26 subjects). As shown in Table 1, no significant differences were found in sex, age, education, duration of disease, TIV, or PSQI score between the CID-HD and CID-LD groups (all *p* > 0.05). The SAS score in the CID-HD group was higher than that in the CID-LD group (*t* = 2.32, *p* = 0.02). The demographic and clinical features were also matched between CID-LD and CID-HD in the second cohort. Pearson’s correlation analysis found that the SDS score was positively associated with duration in the CID-HD group (*r* = 0.31, *p* = 0.03) but not in the CID-LD group. No other significant association was found in the two CID groups.

**TABLE 1:** Demographic and clinical traits for two CID groups.

Characteristic	First Cohort		*T/X^2^*	*P*-value	Second Cohort		*T/X^2^*	*P*-value
	CID-HD (*n* = 26)	CID-LD (*n* = 26)			CID-HD (*n* = 29)	CID-LD (*n* = 24)		
Age	36.12 ± 11.12	41.23 ± 12.37	1.57	0.12	40 ± 10.75	42.21 ± 12.48	0.66	0.51
Gender (M/F)	12/14	9/17	1.95	0.16	8/21	9/15	0.59	0.56
Education	13.50 ± 4.08	12.96 ± 3.84	0.49	0.63	14.89 ± 2.42	14.25 ± 3.22	0.83	0.41
PSQI	14.00 ± 1.72	13.85 ± 1.71	0.32	0.75	13.85 ± 3.20	12.16 ± 3.57	1.79	0.08
SDS	61.81 ± 5.23	46.27 ± 3.18	12.94	<0.001	63.27 ± 7.67	43.18 ± 5.16	9.97	<0.001
SAS	55.54 ± 6.34	52.15 ± 3.86	2.32	0.02	57.54 ± 10.29	43.12 ± 10.89	4.94	<0.001
Duration (Months)	71.92 ± 75.68	45.27 ± 40.81	1.58	0.12	80.04 ± 97.08	90.12 ± 68.64	0.42	0.67
TIV (mm^3^)	1526.62 ± 133.70	1456.41 ± 127.81	1.93	0.06				

*CID, chronic insomnia disorder; CID-HD, CID with high depressive symptom; CID-LD, CID with low depressive symptom; PSQI, Pittsburgh Sleep Quality Index; SDS, Zung self-depression scale; SAS, Zung self-anxiety scale; TIV, total intracranial volume.*

### Comparison of Orbital Frontal Cortex Structure and Function Between Patients With Chronic Insomnia Disorder With High Depressive Symptom and Chronic Insomnia Disorder With Low Depressive Symptom

The right anterior OFC volume in the CID-HD group was decreased compared to that in the CID-LD group (Figure 1B and Table 2). Thus, we used the group difference volume region (right anterior OFC) to construct the OFC FC network. However, we did not find significant group differences in the OFC FC network (using voxel-level *p* < 0.05 and GRF correction *p* < 0.05). Finally, we presented the results without multiple comparison correction, and the potential group difference in the OFC FC network was located in the left inferior occipital gyrus and left insula (Table 2).

**TABLE 2:** The group differences in OFC GM and OFC FC network.

Brain region	BA	Voxel size	MNI coordinates (RAI)			Peak *Z* Score
			*x*	*y*	*z*	
OFC GM (Voxel level *p* < 0.005, Cluster level *p* < 0.05, Cluster size > 100)						
Right anterior OFC	11	267	22	60	−16	−4.21
Right anterior OFC based FC network (Voxel level *p* < 0.05, no multiple comparison correction, Cluster size > 50)						
Left IOG	18	50	−42	−87	−18	−3.87
Left Insula	48	70	−33	9	−18	3.45

*OFC, orbital frontal cortex; FC, functional connectivity; GM, gray matter; BA, Brodmann’s area; IOG, inferior occipital gyrus.*

### Support Vector Machine Classification Results

**FIGURE 2:**
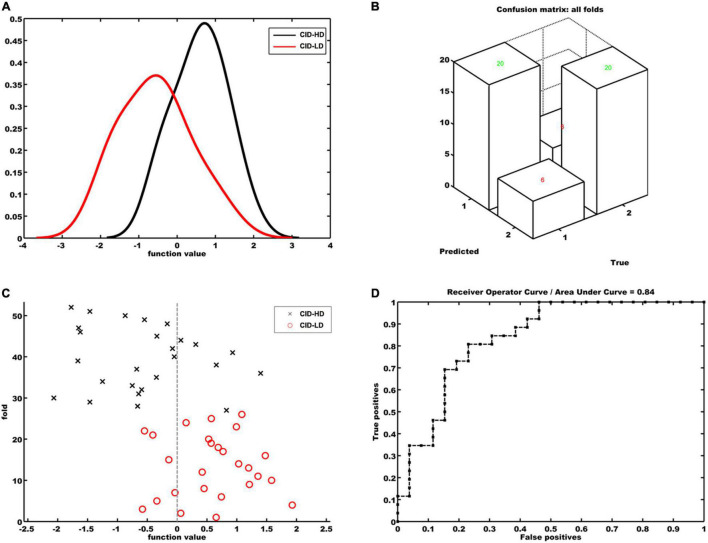
Classification model for discriminating CID-HD from CID-LD patients based on the functional connectivity map of the right anterior orbital frontal cortex. **(A)** Histogram of function values for each group. **(B)** Confusion matrix of all folds. **(C)** Prediction values per fold of the classification model. Positive function values for CID-LD patients indicate true positives. Negative function values for CID-HD participants indicate true negatives. **(D)** Receiver operating characteristic curve (ROC) showing the area under the curve was 0.84. True positives = sensitivity; false-positives = 1 – specificity.

The classification results based on the FC features of the OFC functional network are displayed in Figure 2 (When the significant threshold was as *p* < 0.05 in feature selection). The classification model showed a total accuracy of 76.92% (*p* = 0.0009), the sensitivity of 76.92%, and specificity of 76.92%. The AUC of the classification model was 0.84, and the permutation test showed that the AUC was significant (*p* < 0.001). We also set the GM volume of the OFC as an input feature; however, the SVM model was not able to differentiate the two groups with a total accuracy of 60.38% (*p* = 0.09). The AUC of the GM-based classification model was 0.61, and the permutation *p* was 0.06. Additionally, when we set the significant threshold in feature selection as *p* < 0.01, the classification accuracy for discriminating CID-HD from CID-LD was 67.31% (*p* < 0.01, sensitivity: 65.42%, specificity: 69.20%).

**FIGURE 3:**
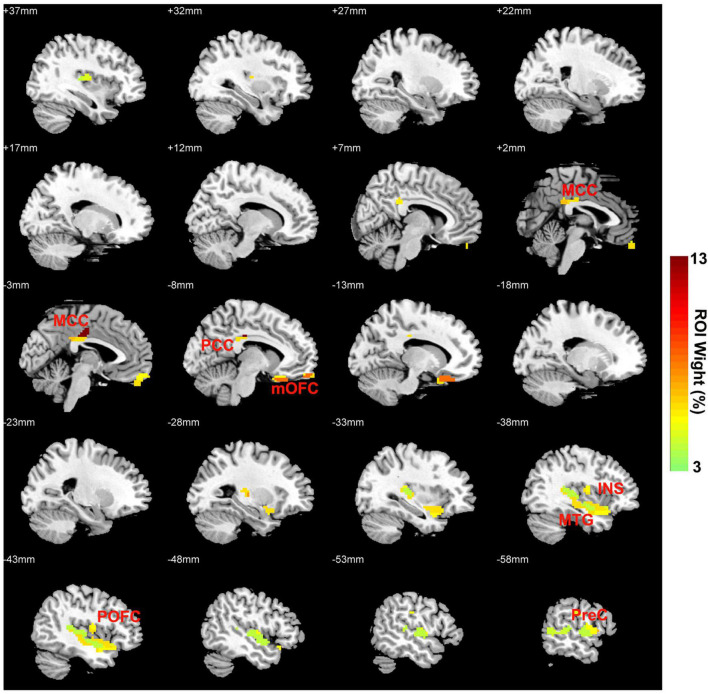
OFC FC network weight images (per ROI) for discriminating CID-HD and CID-LD patients. OFC, orbital frontal cortex; FC, functional connectivity; CID-HD, chronic insomnia disorder with high depressive symptoms; CID-LD, CID with low depressive symptoms; MCC, middle cingulate cortex; mOFC, medial orbital frontal cortex; INS, insula; POFC, inferior par opercularis frontal cortex; PCC, posterior cingulate cortex; MTG, middle temporal gyrus.

The regions contributing to the classification are shown in Table 3 and Figure 3. The first five important regions contributing to the discriminating model were located in the Cingulate_Mid_L (left middle cingulate cortex, MCC), OFCmed_L (left medial OFC, mOFC), Frontal_Inf_Oper_L (left inferior par opercularis frontal cortex, POFC), Cingulate_Post_L (left posterior cingulate cortex, PCC) and Temporal_Mid_L (left middle temporal gyrus, MTG). These brain regions are involved in the reward network (RN, mOFC), salience network (SN, MCC, MOPFC and insula), and default mode network (DMN, PCC, and MTG).

**TABLE 3:** The average ROI weight for classification.

AAL label	ROI name	ROI Wight (%)	ROI Size (Voxel)
33	Cingulate_Mid_L	13.38	28
25	OFCmed_L	6.94	44
11	Frontal_Inf_Oper_L	5.35	13
35	Cingulate_Post_L	4.84	16
85	Temporal_Mid_L	4.56	121
1	Precentral_L	3.98	305
29	Insula_L	3.84	98
34	Cingulate_Mid_R	3.81	14

*AAL, automated anatomical atlas; ROI, region of interest.*

In the verification analysis, the classifier achieved an accuracy of 67.9%, sensitivity of 79.31%, and specificity of 54.17%. The AUC of the classification model was 0.67, and the permutation test showed that the AUC was significant (*p* < 0.001).

## Discussion

Previous findings suggested that patients suffering from insomnia and depression may have a structural deviation in the OFC. Based on these findings, the present study aimed to evaluate whether the altered brain regions of the OFC-based FC network pattern could discriminate CID patients with higher depression from CID patients with lower depression. The results verified our hypothesis that CID-HD and CID-LD could be classified with 76.92% accuracy with the OFC FC pattern classifier, while the RN, SN, and DMN had the highest contribution weights. In addition, the classifier pattern results have been verified by an independent cohort of the CID group. Our findings suggested that OFC FC might be the critical biological feature in the discrimination of higher depression in CID. These findings might provide a potential biomarker for early diagnosis and intervention in CID patients who are comorbid with depression.

The clinical features of the two subgroups of CID showed that the duration of insomnia, but not the severity of insomnia, was associated with the severity of depression only in the CID-HD subgroup. This finding indicated that the duration of insomnia could be a risk factor for depression in CID patients. Accumulating previous brain structure studies have manifested a reduced volume in the OFC in both CID and MDD patients (18, 20, 21, 41, 42). Recently, Cheng et al. investigated sleep duration, brain structure, and depressive problems in 11067 subjects from the Adolescent Brain Cognitive Development study, and the results showed that a higher depressive score in adolescents with low sleep duration was associated with reduced brain regional volume in the lateral and medial OFC (43). According to the automated anatomical labeling atlas 2/3 brain regions of the OFC, eight subdivisions were defined by cytoarchitectonic areas (35, 44). In the present study, we found that the CID-HD group showed reduced OFC gray matter volume in the right anterior OFC, which was mainly located in area 11 defined by Öngür et al. (44) and the medial OFC (BA 13) (35). The anterior OFC has high functional connectivity with the medial and posterior OFC, mainly encoding reward-related information (17, 45). Our previous study found that the FC strength between the right OFC and nucleus accumbens was decreased and associated with depressive symptoms in patients with CID (46). These findings might indicate that CID patients with depression might suffer from dysfunctional reward information encoding in the OFC during insomnia progression, which leads to depressive symptoms.

The results of SVM classification based on OFC FC features showed relatively high accuracy (with both high sensitivity and specificity) for discriminating CID-HD from CID-LD. Previous neuroimaging studies in insomnia have used classification models to discriminate CID patients from healthy controls (16, 47, 48). The regions contributing more to the classifier were the bilateral MCC, left mOFC, left MOPFC, left PCC, left MTG, and left insula. The anterior and medial OFC are involved in the reward network (RN), the MCC, MOPFC, and insula are located in the salience network (SN), and the PCC and MTG are located in the default mode network (DMN) (49–51). Previous studies also reported that abnormal DMN was associated with depressive symptoms in patients with insomnia (52), and abnormal SN was associated with insomnia complaints in patients with MDD (53). According to our findings, the functional connectivity pattern between the RN, SN, and DMN in patients with CID could be a valuable biomarker for identifying subjects who are comorbid with depression.

It should be noted that the between-group differences in anterior OFC functional connectivity did not reach statistical significance. At the uncorrected level, only the left insula could overlap with the seventh contributed region in the machine learning classifier. In addition, the verification analyses of SVM, these classifier pattern of OFC FC could discriminate CID-HD from CID-LD in and dependent CID cohort, and get an accuracy rate of 67.9%. These results were consistent with previous OFC functional connectivity studies of healthy populations with sleep and depressive problems in the HCP dataset (22). Taken together, our study demonstrated the role of the reward network in the pathophysiology of insomnia combined with depression, and suggested that the machine learning approach combined with the OFC FC pattern appears to be more sensitive for CID-HD biomarker detection than traditional mass univariate group comparison.

The current study has several limitations. First, the sample size was small, and future studies could enroll larger sample sizes and multicenter participants to verify the present results. Second, all the patients were medication naïve or washout before neuropsychological testing and MRI scans; however, we could not control for possible confounding effects of recurrent insomnia and history of medication usage. Third, the present study strictly focused on OFC structural changes and detected a significant gray volume alteration in the right anterior OFC in CID-HD, whereas another potential brain region associated with insomnia and depression, such as the amygdala and anterior cingulate cortex, was unexplored (54). Last, CID with anxiety symptoms is another common subgroup, which might be attributed to different biomarkers of depression (55). Future studies should explore potential biomarkers for classifying depression from anxiety in the CID group.

## Conclusion

The present study found that the comorbidity of depression in CID showed reduced region volume in the anterior OFC. Importantly, we demonstrate that the OFC pattern could be a high classifier to detect CID patients with higher depressive symptoms. These findings suggested that machine learning methods can help to identify neurobiological markers to predict depressive symptoms in CID patients based on individual OFC functional connectivity patterns.

## Data Availability Statement

The raw data supporting the conclusions of this article will be made available by the authors, without undue reservation.

## Ethics Statement

The studies involving human participants were reviewed and approved by the Research Ethics Committee of CSPH and AHMU approved this study, and all participants gave written informed consent. The patients/participants provided their written informed consent to participate in this study.

## Author Contributions

CX and ZH contributed to the conception and design of study. LG and RX wrote the manuscript and performed imaging data analysis. JW, XD, XZ, and BZ contributed to the clinical estimate acquisition of imaging data. ZH and DY contributed to revising the manuscript logically for important theoretical content. All authors contributed to the manuscript and have approved the final manuscript.

## Conflict of Interest

The authors declare that the research was conducted in the absence of any commercial or financial relationships that could be construed as a potential conflict of interest.

## Publisher’s Note

All claims expressed in this article are solely those of the authors and do not necessarily represent those of their affiliated organizations, or those of the publisher, the editors and the reviewers. Any product that may be evaluated in this article, or claim that may be made by its manufacturer, is not guaranteed or endorsed by the publisher.
